# Posterior shoulder tightness; an intersession reliability study of 3 clinical tests

**DOI:** 10.1186/s40945-020-00084-w

**Published:** 2020-07-29

**Authors:** Kevin Hall, Jeremy Lewis, Ann Moore, Colette Ridehalgh

**Affiliations:** 1 Western Sussex Hospitals NHS Trust, Lyndhurst Rd, Worthing, West Sussex BN11 2DH UK; 2 School of Health and Social Work, University of Hertfordshire, Hatfield, Hertfordshire AL10 9AB UK; 3 Therapy Department, Central London Community Healthcare National Health Service Trust, London, UK; 4 Department of Physical Therapy & Rehabilitation Science, College of Health Sciences, Qatar University, Doha, Qatar; 5 School of Health Sciences, University of Brighton, Room 203, Aldro Building, 49 Darley Road, Eastbourne, East Sussex BN20 7UR UK; 6 School of Health Sciences, University of Brighton, Robert Dodd Building, 49 Darley Road, Eastbourne, East Sussex BN20 7UR UK

## Abstract

**Background:**

Although posterior shoulder tightness (PST) has been associated with shoulder pathology and altered glenohumeral joint kinematics, uncertainty remains regarding its cause and definition. To understand the efficacy of treatments for PST, it must be possible to identify people with PST for the purposes of research and clinical decision-making. Clinical tests for PST must demonstrate acceptable levels of measurement reliability in order to identify the condition and to evaluate the response to intervention. There is currently a lack of research describing intersession reliability for measures of PST. The aim of this study was to quantify the inter-session reliability for three clinical tests used to identify PST over a 6–10 week interval.

**Methods:**

A convenience sample of 26 asymptomatic adult participants (52 shoulders) were recruited from a university setting over a five-month duration. Participants attended the human movement laboratory for measurement of glenohumeral joint internal rotation, horizontal adduction and low flexion on two occasions separated by an interval of 6–10 weeks.

Intra-class correlation coefficients were calculated from the mean square values derived from the within-subject, single factor (repeated measures) ANOVA. Test-retest measurement stability was evaluated by calculating the standard error of measurement and the minimum detectable change for each measurement.

**Results:**

All 3 tests demonstrated good intersession intra-rater reliability (0.86–0.88), and the standard error of measurement (95%) were 7.3° for glenohumeral horizontal adduction, 9.4° for internal rotation, and 6.9° for low flexion. The minimum detectable change for glenohumeral horizontal adduction was 10.2°, internal rotation was 13.3°, and low flexion was 9.7°.

**Conclusion:**

In this population of people without symptoms, the 3 measures of PST all demonstrated acceptable inter-session reliability. The standard error of measurement and minimum detectable change results can be used to determine if a change in measures of PST are due to measurement error or an actual change over time.

## Background

### Introduction

Shoulder pain has been described as the third most common musculoskeletal disorder presenting to general practitioners [1]. Estimates of 1-year prevalence range from 7% to over 20% [2]. Prognosis is often poor, with 50% of shoulder pain persisting at 3-year follow up [3], and ongoing shoulder pain may severely affect an individual’s perception of their general health and wellbeing [4].

Posterior shoulder tightness (PST) has been proposed as an important physical impairment in the management of the athletic shoulder [5–7] and increasingly in the non-sporting shoulder [8, 9]. Many authors recommend treatment of the posterior shoulder as part of the treatment algorithm for the management of shoulder pain [10, 11], and many RCT’s investigating the impact of exercise on shoulder pain have included a ‘posterior shoulder’ intervention as part of their exercise program [12–14].

### Clinical tests for posterior shoulder tightness

If PST is a cause of shoulder pain or a barrier to recovery, it must be identifiable in a clinical setting. The clinical tests used to identify PST are important for two reasons, firstly, to identify PST, through side-to-side differences in shoulder ROM, and secondly to detect measurable change in PST following intervention. In order to be able to do these two things, the clinical tests must demonstrate acceptable levels of reliability. Reliability has been defined as ‘the extent to which a measurement is consistent and free from error’ [15]. All measurements contain a component of error, for example, the measured value of a movement is a product of the true value and the error of measurement. The true value of a measure can fluctuate over time. Inter-session reliability provides information on the error of measurement of a test, and, on how the measure fluctuates over time.

PST has historically been defined through side-to-side differences in measures of horizontal adduction and internal rotation [5, 16]. A third test was proposed by Borstad and Dashottar [17], who performed a cadaveric study on 8 fresh cadaveric shoulders to determine the strain on the structures of the posterior shoulder with 5 simulated clinical tests or movements of the humerus. They found greatest strain on the posterior capsule using a position of 60° flexion and internal rotation, and concluded that this test was valid for the purpose of evaluating glenohumeral joint posterior capsule flexibility. The authors named this movement ‘low flexion’ [17] and recommended its use in the assessment of PST.

Based on the current body of evidence the three clinical tests that may be used as a measure of PST are:

1. Glenohumeral horizontal adduction in supine (HorAdd)
2. Shoulder internal rotation at 90-degrees abduction (GHj-IR)
3. Low flexion, shoulder internal rotation at 60 degrees of shoulder flexion (LF)

### Defining posterior shoulder tightness

PST has been defined in terms of reduced glenohumeral joint internal rotation (GHj-IR) and, or glenohumeral horizontal adduction (HorAdd) compared to the contralateral shoulder [18, 19]. The use of the term, ‘posterior shoulder tightness’ is prevalent in the literature but its definition remains unclear (Table 1), and it is frequently referred to without being defined [23–25]. Some definitions imply an anatomical source responsible for the deficit in range [20, 21], however, making assumptions about the structure responsible for the deficit is misleading, and cannot be confirmed either through clinical examination or medical imaging. Myers et al. [22] explained that, ‘PST is typically measured by passively assessing the amount of humeral internal rotation deficit [ …] or horizontal adduction’ (pg 1923), but for the purpose of their study, ‘operationally defined PST as the percentage difference in the amount of horizontal adduction’. Many studies have used a deficit of either internal rotation [9] or horizontal adduction [8] or low flexion [26] to define PST for the purposes of inclusion into interventional studies or correlational studies, and considered a side-to-side difference ranging from 7° [26] to 20° [27] as an indication that PST is present. This lack of clarity over the definition of PST may result in heterogeneity of studied populations. Defining PST more clearly is important for the purposes of research and clinical practice. Based on cadaveric studies [17, 28], reliability data [29, 30] and its historical origins [5, 16], we have defined PST in terms of the three measures described above;

A side-to-side difference of 10° or more in 2 out of 3 clinical tests (GHj-IR, HorAdd and LF) or a difference of 20° or more in a single test.

**Table 1:** Definitions of Posterior Shoulder Tightness

Posterior Shoulder Tightness (PST)	
Salamh et al. [20]	‘PST has been defined as a limitation of the extensibility within the posterior soft tissue of the shoulder including both contractile and non-contractile elements as well as osseous changes as seen in the form of humeral torsion within the overhead athlete through training adaptations’ (pg 179)
Mine et al. [19]	‘PST is clinically measured by passive shoulder horizontal adduction with the scapula stabilized in supine or side-lying. GIRD is generally characterized as concurrent deficits of internal rotation (IR) and total arc of motion in the dominant side’ (pg 294)
Borstad et al. [18]	‘Posterior shoulder tightness is most often assessed by quantifying horizontal adduction (HAD) or supine glenohumeral joint (GHJ) internal rotation (IR) range of motion’ (pg 875–876)
Dashottar et al. [21]	‘The internal rotation (IR) loss is attributed to osseous and soft tissue adaptations and is referred to as posterior shoulder tightness (PST)’ (pg 499)
Myers et al. [22]	‘Operationally defined PST as the percentage difference in the amount of horizontal adduction’ (pg 1923)

Several studies have described the reliability of GHj-IR, HorAdd and LF. Most of these studies have described intraclass correlation coefficients (ICC) values obtained through intra-session testing;

1. HorAdd intra-rater, intra-session ranging from 0.91–0.94 [22, 30] and intra-rater, inter-session ranging from 0.74–0.93 [30, 31]
2. GHj-IR intra-rater, intra-session ranging from 0.72–0.99 [29, 32] and inter-rater, inter-session ranging from 0.43–0.47 [33]
3. LF intra-rater, intra-session ranging from 0.90–0.95 [18, 21]

One of the major gaps in the literature relating to the measurement of PST is the lack of intersession reliability data. The majority of studies collected their data within a single session and those that have looked at intersession reliability either do so over an insufficient interval to represent a physiotherapy treatment interval (e.g. [22], 3–7 days) or use measurement techniques more susceptible to random error [31]. The aim of the study was to establish intersession ICC values over a 6–10 week duration in order to calculate standard error of measurement (SEM) and the minimum detectable change (MDC) of 3 measures of PST over a treatment period consistent with a typical episode of physiotherapy. This study was conducted in preparation for a clinical trial investigating the impact of treating PST in patients with rotator cuff related shoulder pain (RCRSP) (ClinicalTrials.gov Identifier: NCT02598947).

## Methods

### Participants

Institutional ethical approval was provided by the relevant ethics review board prior to commencing the study. A convenience sample of 26 asymptomatic adult participants were recruited from the local university setting over a five-month duration from February to August 2014. The sample had a mean age of 35(SD = 12.7) years, mean weight of 72(SD = 11.3) Kg, and mean height of 171(SD = 9.4) cm, 88% were right handed and 65% were female. Participants were contacted by telephone and screened for the exclusion criteria below. All participants completed a review-board approved informed consent agreement before participation. Participants were excluded from the study if they reported pain in the neck, upper back or either upper limb in the preceding 2 months, had previous surgery or fracture to the shoulder or neck, had known osteoarthritis of shoulder, were unable to lie supine, were pregnant or epileptic, or had systemic inflammatory conditions. Participants were asked to avoid high intensity sporting activity involving the shoulder in the 24 h prior to all measurements. A sample size calculation was performed according to guidance provided by Walter et al. [34], where ICC value Po = 0.7 (minimum acceptable ICC value) and P1 = 0.8 (predicted ICC value). A sample size of 39 was recommended with a drop out rate of 20% from initial to subsequent testing generated a target recruitment of 47 shoulders (24 participants).

### Procedures

**Fig. 1:**
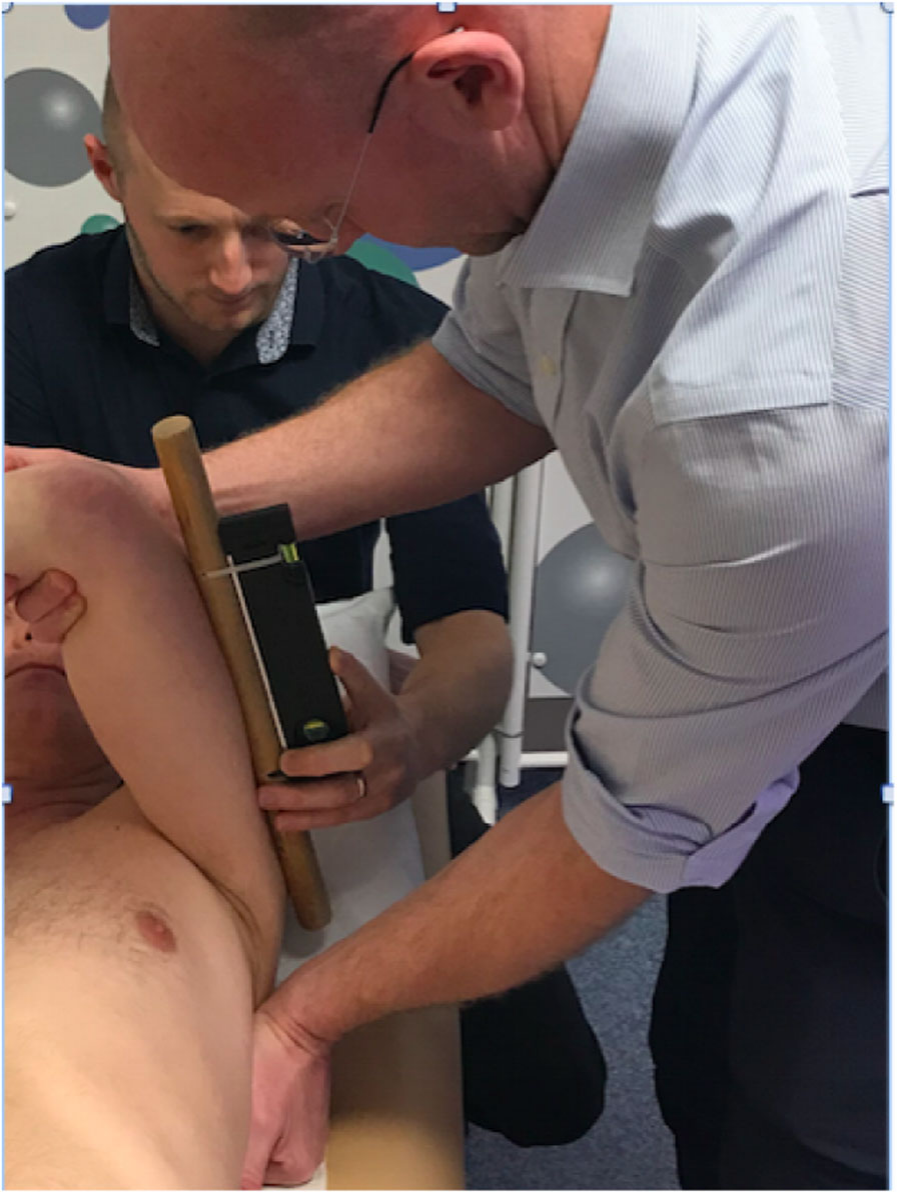
Glenohumeral joint horizontal adduction (HorAdd)

**Fig. 2:**
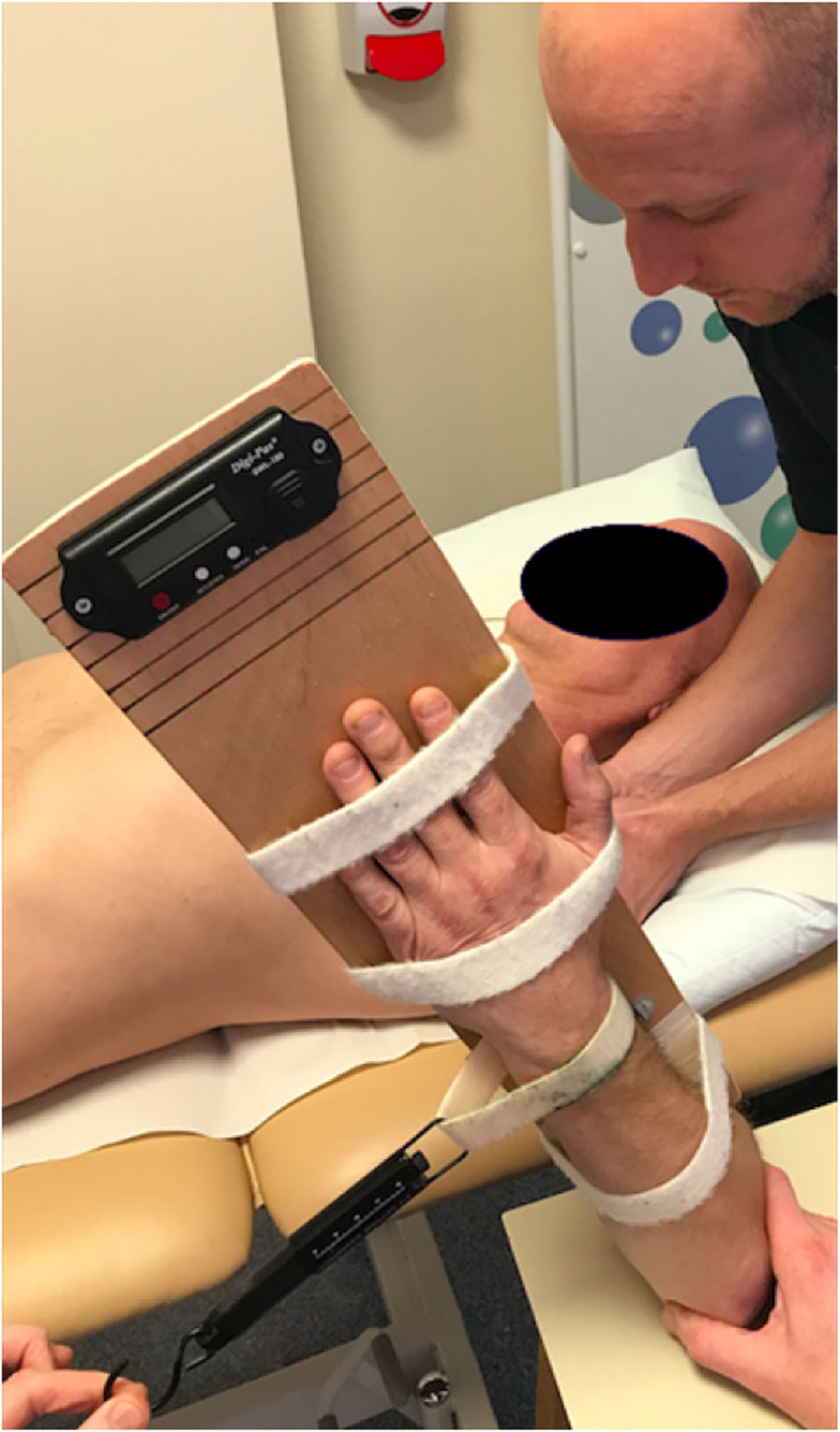
Glenohumeral joint internal rotation in abduction (GHj-IR)

**Fig. 3:**
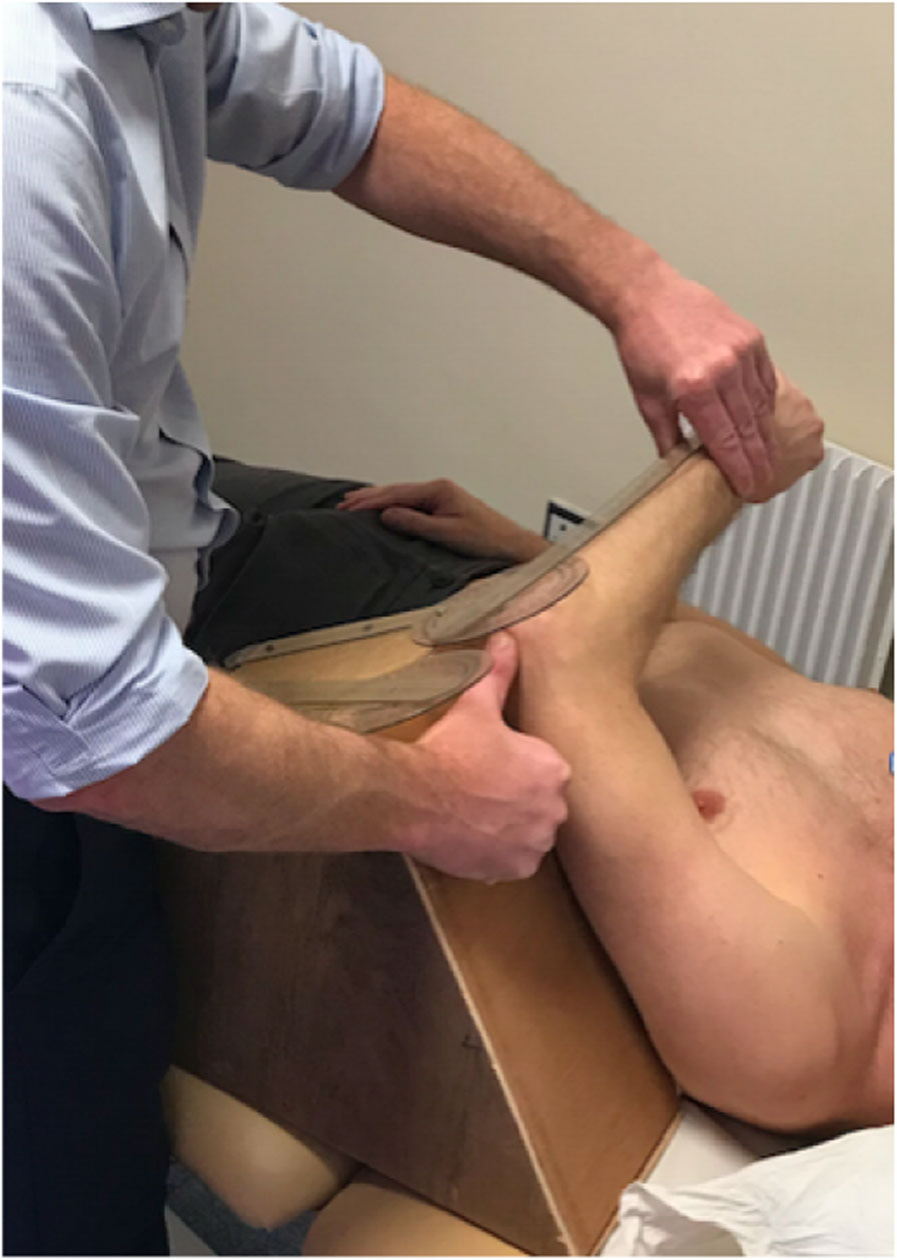
Low flexion (LF)

Participants attended the human movement laboratory for measurement on two occasions separated by an interval of 6–10 weeks. Three clinical tests were performed on both shoulders at each visit. All measurements were performed by the same assessor (KH) who had 15 year’s experience working in musculoskeletal physiotherapy, and who was blinded to the measurement recorded by a trained assistant (see Figs. 1, 2 and 3).

Prior to each measurement the equipment was checked for damage and the digital inclinometers were calibrated. The purpose of the study was explained to the participants and a standardized warm-up and measurement protocol was implemented. The order of testing for each participant and for each shoulder was drawn at random at each visit by the lead investigator (KH).

**Horizontal adduction (HorAdd)** was measured in supine by stabilizing the scapula and taking the arm into horizontal adduction, to the end of the available passive range, whilst maintaining 90-degrees elevation and neutral rotation of the glenohumeral joint. A research assistant measured the angle formed by the upper arm and the horizontal using a digital inclinometer (Digi-Pas DWL-200 Digital Level, Tarax Technology Ltd., Dundee, UK, accurate to 0.1 degrees). Measurements were rounded to the nearest degree (see Fig. 1).

**Internal rotation in abduction (GHj-IR)** was measured using a measurement brace that fixed the elbow in 90 degrees flexion (Fig. 2). A strut was used to maintain 90 degrees abduction during the measurement and a standardized force of 1 kg of overpressure was applied through a suspension scales. An inclinometer (Digi-Pas DWL-180 Digital Level, Tarax Technology Ltd., Dundee, UK, accurate to 0.1 degrees) fixed onto the measurement brace was used to record the angle of rotation. Measurements were rounded to the nearest degree.

**Low flexion (LF)** involves the measurement of glenohumeral joint internal rotation in a position of 60-degrees flexion. Low flexion is a multi-axis measurement and so the use of a single axis inclinometer would create cross-axis error. For this reason the measurement was performed using a purpose built wedge and mounted long arm goniometer (30 cm). The goniometer was securely mounted at right angles to the 60-degree wedge. The arm to be tested was held in 60-degrees flexion by the purpose built wedge and the participant’s shoulder was then internally rotated using the forearm together with the free arm of the goniometer to the end of the available passive range (see Fig. 3). All measurements were rounded to the nearest degree. The long arm goniometer was calibrated by manufacturers with the International Standards of Measurement System (ISOM).

Two measurements were taken for each test with a 30 s interval and if the difference between the two readings was greater than 5-degrees, a third measurement was taken and the average of the two closest values recorded. If the first two measurements were different by 5-degrees or less, the average of these two measures was recorded [35]. All three measurements on both arms were repeated with a 6–10 week interval.

### Data analysis

Collected data was transferred to SPSS Version 15.0 (SPSS Chicago, Illinois) for Windows™ was used for the analysis. Descriptive data were calculated for each measure, including mean angles of measurement and standard deviations (SD). The ICCs were calculated from the mean square values derived from the within-subject, single factor (repeated measures) ANOVA. Test-retest measurement stability was evaluated by calculating the SEM as the square root of the subject’s mean square error.

The MDC was calculated for the inter-session measurements using the formula: MDC = 1.96 x√2 x SEM. MDC is calculated in terms of a confidence of predication. So MDC95 describes the minimum amount of change in a patient’s score that is due to real change and not the error of measurement or natural fluctuation in 95% of cases.

## Results

The datasets supporting the conclusions of this article are included within this article (and its additional file). ICC values were calculated for each of the three tests and the stability measures of SEM and MDC were calculated (Table 2). All 3 tests demonstrated good intersession intrarater reliability (ICC 0.86–0.88). SEM ranged from 3.5–4.8 degrees and MDC ranged from 9.7–13.3 degrees. The SEM (95%) was calculated (+/− 1.96 x SEM) for each of the three measures of PST from the inter-session intra-rater reliability data;

- 7.3° for horizontal adduction (SEM = 3.7°)
- 9.4° for internal rotation in abduction (SEM = 4.8°)
- 6.9° for low flexion (SEM = 3.5°)

**Table 2:** Intra-rater reliability (ICC) and test-retest measurement stability (SEM and MDC)

	ICC value (95% confidence interval)	Standard error of measurement (SEM) (Degrees)	Minimum Detectable Change (MDC) (Degrees)
Horizontal adduction	0.883 (0.802–0.932)	3.7	10.2
Internal rotation in 90 degrees abduction	0.869 (0.753–0.928)	4.8	13.3
Low Flexion	0.857 (0.745–0.919)	3.5	9.7
• Two-way mixed effects model where people effects are random and measures effects are fixed. • Type A intraclass correlation coefficients using an absolute agreement definition. • The estimator is the same, whether the interaction effect is present or not. • Minimum Detectable Change (MDC) $=1.96\times \sqrt{2}\times SEM$ [36]			

## Discussion

PST has been implicated in many shoulder conditions and can be identified using three clinical tests: HorAdd, GHj-IR and LF. To this authors knowledge this is the first study to describe intersession reliability data for the 3 clinical tests used to identify PST over a time frame consistent with a physiotherapy treatment episode.

ICC’s yield a value between 0.0 and 1.0, where anything above 0.75 is considered to represent ‘good’ reliability [15]. What constitutes ‘good’, however, really depends on what the test is required to do. One of the problems with ICC values is interpretation and practical application. For example an ICC = 0.87 has no practical meaning. The SEM has direct practical applications and is calculated using the ICC values. The SEM quantifies measurement error in the same units as that of the test. An SEM of 3.7° for HorAdd, suggests that if a difference of more than 3.7° is detected, we can be confident in 68% (1 SEM = SEM68%) of cases that this difference is due to a real difference and not just error of measurement or natural fluctuation. If, for example, a value of 30° HorAdd is measured, the SEM95% suggests that the true value of HorAdd in that shoulder lies between 30° +/− (1.96 × 3.7°). Thus the true range lies between 23 and 37° in 95% of cases. In response to treatment, the MDC would suggest that if a change in HorrAdd of more than 10.2° was detected this would reflect a true change in 95% of cases.

The only other study evaluating intersession reliability over a time frame consistent with a physiotherapy treatment episode was conducted by Borstad et al. [31], who described intersession reliability values for HorAdd and GHj-IR over an 8–12 week interval. They described ICC values of 0.74 and 0.79 for supine horizontal adduction and internal rotation in abduction respectively in asymptomatic participants. Our higher ICC values of 0.88 and 0.87 may be a result of the method of assessment utilized. In this study measures were taken to minimize random error, such as the use of a standardized force of overpressure, inclinometry instead of goniometry and implementation of a standardized warm-up protocol.

The proposed definition of PST, incorporates the 3 clinical tests commonly described in this reliability study:

A side-to-side difference of 10° or more in 2 out of 3 clinical tests (GHj-IR, HorAdd and LF) or a difference of 20° or more in a single test.

Using several tests to identify PST may enable clinicians to overcome the inherent weaknesses or variability of individual tests. There is limited data available on the correlation of side-to-side differences between these tests, so it is not known if a deficit in LF correlates with a deficit in HorAdd. Each test, however, is likely to influence the tissues of the shoulder in different ways, and be influenced by bony architecture in different ways. Making a decision to identify PST based on the outcome of a cluster of tests may better reflect the complex anatomy of the posterior shoulder and the multiple layers of connective tissue and muscle acting as potential passive restraint to movement. The use of clustering clinical tests to identify conditions has been used extensively in the literature, for example to identify sacroiliac pain [37], headaches [38] and cervical myelopathy [39]. Although the definition of PST does not imply that a specific anatomical structure is responsible for the deficit in range, evidence does exist from cadaveric [17] and in vivo experimental studies [21] that movements involving horizontal adduction and internal rotation generate strain in the structures of the posterior shoulder. All three tests used in this study exhibit strong face validity as they all conform to the original concept of PST being identified as a side-to-side deficit involving internal rotation and horizontal adduction.

The SEM95% for the three measures of PST described in this study range from 6.9–9.4°, which lies within this 10° range described in the definition. Therefore if a side-to-side difference is identified that exceeds the SEM95%, it may be concluded that this is a true difference and not attributable to error in 95% of cases.

### Study limitations

One limitation of this study is that it was performed on people without pain. Application of the test in clinical practice will normally involve a comparison between a person’s painful shoulder and their non-painful shoulder. As a result establishing the inter-session reliability of the tests in people without pain is relevant to its use in clinical practice. Performing the tests on a person’s painful shoulder might result in increased random error as a result of apprehension, bracing or guarding. Intra-session reliability studies in symptomatic participants have demonstrated high levels of reliability suggesting that the presence of pain does not automatically result in greater random error. Borstad et al. [18] described higher ICC values in Intra-session assessment of LF in symptomatic participants (0.94) compared with asymptomatic participants (0.90). If random error can be minimised in intra-session reliability studies with symptomatic participants it is still possible that there might be more natural fluctuation over time in measures of PST in the presence of pain. This would only be identified through inter-session reliability studies in symptomatic participants. In their inter-session reliability in symptomatic and asymptomatic participants, Borstad et al. [31] described higher ICC values in symptomatic participants (0.79) compared with asymptomatic participants (0.74). Measures of PST, therefore, seem to be stable over time in symptomatic and asymptomatic populations.

## Conclusion

Based on the findings of this study the 3 measures of PST have demonstrated good inter-session reliability in asymptomatic participants. A definition for PST has been proposed which is partly informed by the findings of this reliability study.

## Supplementary information

**Additional file 1.** Supplementary information; dataset for shoulder measurements.

## Data Availability

The datasets analysed during the current study available from the corresponding author on reasonable request.
